# Statistical reporting of metabolomics data: experience from a high-throughput NMR platform and epidemiological applications

**DOI:** 10.1007/s11306-019-1626-y

**Published:** 2019-12-10

**Authors:** Stefan Mutter, Carrie Worden, Kara Paxton, Ville-Petteri Mäkinen

**Affiliations:** 1 Computational Systems Biology Program, Precision Medicine Theme, South Australian Health and Medical Research Institute, Adelaide, Australia; 2 Folkhälsan Institute of Genetics, Folkhälsan Research Center, Helsinki, Finland; 3 Abdominal Center Nephrology, University of Helsinki and Helsinki University Hospital, Helsinki, Finland; 4 Research Program for Clinical and Molecular Metabolism, Faculty of Medicine, University of Helsinki, Helsinki, Finland; 5 School of Pharmacy and Medical Sciences, University of South Australia, Adelaide, Australia; 6 Hopwood Center for Neurobiology, Lifelong Health Theme, South Australian Health and Medical Research Institute, Adelaide, Australia

**Keywords:** NMR, Reporting, Summary statistics, Integration, Meta analysis, Metabolic profiles

## Abstract

**Introduction:**

Meta-analysis is the cornerstone of robust biomedical evidence.

**Objectives:**

We investigated whether statistical reporting practices facilitate metabolomics meta-analyses.

**Methods:**

A literature review of 44 studies that used a comparable platform.

**Results:**

Non-numeric formats were used in 31 studies. In half of the studies, less than a third of all measures were reported. Unadjusted P-values were missing from 12 studies and exact P-values from 9 studies.

**Conclusion:**

Reporting practices can be improved. We recommend (i) publishing all results as numbers, (ii) reporting effect sizes of all measured metabolites and (iii) always reporting unadjusted exact P-values.

**Electronic supplementary material:**

The online version of this article (10.1007/s11306-019-1626-y) contains supplementary material, which is available to authorized users.

## Introduction

Research data can be shared at multiple levels, as raw measurements (e.g. NMR or MS spectra of blood or urine samples), as pre-processed intermediate results (metabolite concentrations for each individual) or as summary statistics (aggregate associations between metabolites and diseases). For raw data, the diversity of analytical workflows is a challenge and we refer to the comprehensive review by the COMETS consortium for further information (Playdon et al. 2019). For metabolic concentrations from individuals, legal and ethical commitments may make sharing difficult in human studies. Consequently, meta-analysis of summary statistics is a common approach in biomedical research. Here, we focus on the statistical reporting with special emphasis on facilitating meta-analyses.

We investigated the reporting practices of ^1^H NMR metabolomics data from a single high-throughput platform (Soininen et al. 2009). The pipeline is built on a highly standardized experimental setup that yields over 200 lipid and metabolite measures from human serum samples. Every researcher receives an identical data spreadsheet. For this reason, the differences in reporting are due to the choices of the authors without being confounded by the technical properties of the analytical platform.

We report findings on the coverage and type of statistics reported in 44 different peer-reviewed papers published in high-quality journals. As metabolomics data are expanding rapidly in clinical and epidemiological studies, we expect our results to help people ensure that the wealth of knowledge can be replicated and re-used effectively and in an unbiased manner.

## Materials and methods

**Fig. 1:**
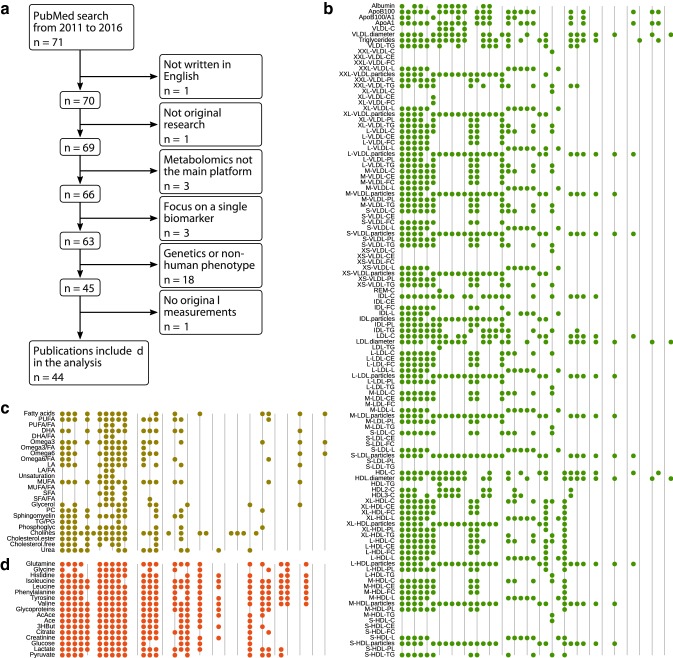
Selection criteria for publications that were included in the analysis (**a**) and the binary heatmap of available summary statistics from 44 research papers organized by the type of experiment (**b**–**d**). Each column represents a paper and each row shows the availability of summary statistics for a metabolic measure. The results for the lipoprotein window (**b**), lipid window (**c**) and low-molecular weight molecules window (**d**) are shown

We conducted a literature search for all peer-reviewed articles that reported results from a single NMR metabolomics platform (Soininen et al. 2009) between January 2011 and August 2016. Publication lists were obtained from PubMed using all three main platform authors as keywords (‘Kangas AJ’ and ‘Soininen P’ and ‘Ala-Korpela M’). During the time period, these authors were always included in papers that used the NMR data as a standard practice. The initial pool contained 71 publications. Figure 1a describes the selection process of eligible studies from the initial pool. A total of 44 papers were included for further analyses (Supplement Table S1).

We extracted the list of metabolites that were routinely reported to all end-users, and the types of summary statistics that were available for each metabolite from the publications. To assess the number of reported metabolites, we then chose the type of statistic that covered the largest number of metabolites as the primary profile. If multiple profiles covered the largest number of metabolites, we preferred interventional over longitudinal and over cross-sectional profiles. If there were still multiple possible primary profiles, we chose the primary profile that was generated from a larger sample size.

The NMR metabolomics platform comprised three “molecular windows” for (i) lipoprotein subclasses, (ii) low-molecular weight metabolites in an intact serum sample, and (iii) the composition of lipid species after chemical lipid extraction from the same sample (Soininen et al. 2009; Würtz et al. 2017). The first two windows were always included, whereas the third window of lipid species was optional. Small modifications were made between 2011 and 2016 to the platform affecting the number of reported metabolites. The latest version (Würtz et al. 2017) included 228 metabolite measurements. The lipoprotein measures covered 14 subclasses and each subclass was reported as concentrations and as a percentage of total lipids. We did not make a distinction between concentration or percentage—either was a sufficient indicator of availability and only counted once if both were available. Therefore, we counted the presence of 158 distinct metabolite measures. An earlier version of the platform (Inouye et al. 2010) also reported 14 measures that were altered or replaced in later versions. Altogether, we created a master list of 172 metabolite measurements.

## Results and discussion

We included 44 studies, of which 21 we classified as cross-sectional designs, 12 as cross-sectional designs with a longitudinal clinical endpoint (NMR analysis was performed at baseline only) and 11 as longitudinal studies with at least two NMR measurements. The median number of participants was 738 (IQR 3537; min 12; max 10,083). We found 24 different cohorts in the 44 studies. Most of the cohorts were from Finland (19 cohorts) and five were from Europe. All but two studies included both men and women. Most papers were under an open access scheme (35 out of 44). We identified three important issues: (i) non-numerical result formats, (ii) selective reporting of a few metabolites instead of the full profile and (iii) different indicators for statistical evidence between studies.

Regarding the result format, we found that only 13 out of 44 primary profiles were included in a spreadsheet or a text document in such a form that the results could be easily converted to numbers. Usually, the results were either embedded in the main text (24 out of 44) or as a PDF supplement (7 out of 44). Six studies published their primary profile in a figure. Non-numeric results formats can cause technical problems and typing errors if the values cannot be copied directly or read in by a machine interface.

Most of the results presented measurements for a subset of metabolites only. The median number of measures reported was 36 (IQR 44), which was substantially fewer than the maximum of 172 in the master list. This means that incomplete profiles were the norm rather than the exception (Fig. 1b–d). Selective reporting may prevent the re-use of summary statistics. From a meta-analysis perspective, it is critical to report metabolites that are not showing a statistically significant signal, as they will contribute to the overall meta-statistic. Furthermore, it is difficult for readers to assess the role of multiple testing if only a subset of metabolic data is reported (presumably the authors would have screened all the measures since they are delivered in a single Excel file). There is a great temptation to break a single metabolomics study into several manuscripts to boost publication records, however, such practice may not fit well with the nature of omics data and systems-based interpretation.

The choice for a descriptive statistic depends on the study design and whether the outcome is continuous or categorical. Therefore, it is challenging for all studies to use the same statistical test. Of note, 9 out of the 44 publications reported means and standard deviations, 1 profile reported means only, 3 reported mean differences, 1 reported medians, 9 focused on regression coefficients, 6 reported correlation coefficients, 8 reported either hazard or odds ratios, 2 studies reported percentage changes, 2 reported changes normalized by standard deviations, 1 study reported P-values (of correlation coefficients) only, 1 profile reported the percentage change with respect to the interquartile range and 1 profile reported the area under the curve. Therefore, the descriptive statistics were diverse, which made it difficult to assign mutually comparable effect sizes in meta-analysis and other integrative settings.

Regardless of the descriptive statistic, the *P* value is universally comparable between studies, which makes it an appealing report item for integrative analyses. Two publications out of 44 did not include P-values or confidence intervals for the primary profile, 3 reported confidence intervals only and 4 papers reported only thresholds for P-values. In several studies, adjustments for multiple testing were conducted. Bonferroni adjustment or its variants were applied in 22 papers and 6 papers used the false discovery rate (one study used both adjustments). Both unadjusted and adjusted P-values were reported for 24 profiles. Unadjusted P-values were reported exclusively for 8 profiles, and adjusted P-values exclusively for 3 profiles. The unadjusted P-value is preferred here as there are multiple existing methods to combine P-values from multiple datasets (Blettner and Schlattmann, 2005). Also, P-values should be reported as exact numbers when possible rather than providing an upper limit (i.e. P < 0.05 should be reported as P = 0.0034).

Most study cohorts were from Finland, which means that our source material had limited global coverage. But the articles were published in the top peer-reviewed international journals within their target disciplines and the authors included leading international experts in epidemiology and medicine. For this reason, our results are likely to reflect the reporting practices in metabolomics studies of human cohorts in general.

We investigated how metabolomics data were reported within a subset of papers that used the same NMR metabolomics platform. Importantly, the restricted scope enabled us to focus on the reporting of the results without confounding from technological changes. In summary, we observed a lack of consistency on how the results were reported, which had a negative impact on our ability to re-use the summary statistics for integrative analyses. We recommend the authors of future studies to report both the unadjusted P-value and the effect size regarding the primary outcome for all metabolites, and to include these results as a separate spreadsheet supplement if the journal policy allows it.

## Electronic supplementary material

Below is the link to the electronic supplementary material.

Supplementary material 1 (XLSX 9 kb)
